# Antiviral effect of alkaloids-free Ephedra Herb extract on respiratory syncytial virus infection

**DOI:** 10.3389/fphar.2024.1410470

**Published:** 2024-07-05

**Authors:** Aya Fujikane, Ryosuke Fujikane, Sumiko Hyuga, Yusuke Sechi, Tetsuya Hiyoshi, Atsuhiko Sakamoto, Akinori Nishi, Hiroshi Odaguchi, Kenji Hiromatsu, Yukihiro Goda, Yoshizumi Ishino, Shigeki Nabeshima

**Affiliations:** ^1^ Department of General Medicine, Faculty of Medicine, Fukuoka University, Fukuoka, Japan; ^2^ Department of Physiological Science and Molecular Biology, Fukuoka Dental College, Fukuoka, Japan; ^3^ Oral Medicine Research Center, Fukuoka Dental College, Fukuoka, Japan; ^4^ Oriental Medicine Research Center, School of Pharmacy, Kitasato University, Tokyo, Japan; ^5^ TSUMURA Advanced Technology Research Laboratories, Tsumura & Co., Ibaraki, Japan; ^6^ Department of Microbiology and Immunology, Faculty of Medicine, Fukuoka University, Fukuoka, Japan; ^7^ National Institute of Health Sciences, Kawasaki, Kanagawa, Japan; ^8^ Department of Bioscience and Biotechnology, Graduate School of Bioresource and Bioenvironmental Sciences, Kyushu University, Fukuoka, Japan

**Keywords:** Ephedra Herb, ephedrine alkaloids-free Ephedra Herb extract (EFE), respiratory syncytial virus, G protein, maoto

## Abstract

Respiratory syncytial virus (RSV) is a major cause of respiratory tract infection in children. Despite decades of efforts, no effective therapies are available. We recently reported that extracts of Ephedra Herb and Cinnamon Bark interacted with the G attachment protein of RSV to inhibit infectivity. The present *in vitro* study aimed to investigate the antiviral effect of ephedrine alkaloids-free Ephedra Herb extract (EFE), which is characterized by free of harmful effects of ephedrine alkaloids in Ephedra Herb, on experimental RSV infection. Infection of RSV into A549 cells simultaneously with EFE resulted the significant reduction of RSV RNA, viral protein, and viral titers after the incubation of the cells. We found that RSV attachment to the cell surface was inhibited both in the presence of EFE and when RSV particles were pre-treated with EFE. We also found that EFE specifically interacted with the central conserved domain of RSV G protein by surface plasmon resonance, demonstrating that specific binding of G protein to the cellular receptor was inhibited by EFE. Another mechanism was found in which a higher concentration of EFE inhibited the viral load immediately after the viral entry into host cells, suggesting the inhibition of viral RNA replication. These results demonstrate that EFE worked against RSV infection through multiple antiviral mechanisms, a unique feature of this crude drug extract.

## Introduction

Respiratory syncytial virus (RSV) is known as a pathogen that causes acute respiratory tract infection in people of all ages. Most people are infected in infancy, and some of them become severely ill with lower respiratory tract infection. Approximately 100,000 people die annually from this disease, mainly children in developing countries, and millions require hospitalization and treatment (Shi et al., 2017; Young and Smitherman, 2021). Palivizumab and nirsevimab, humanized monoclonal antibodies, are only available for the prophylaxis in children, but with high risk, and various vaccines and monoclonal antibodies are being tested in clinical trials (Griffin et al., 2020). Given the large number of patients with RS virus infection worldwide, the development of oral drugs based on small molecules is urgently needed, as is also the case for Ebola and COVID-19 (Tang et al., 2019).

RSV belongs to the Orthopneumovirus genus of the Pneumoviridae and has a negative-sense, single-stranded RNA genome. It has two outer spike proteins on the envelope: G which is responsible for the functions of adhesion to the receptor and F for fusion to the cell membrane. We recently reported that herbal extracts of Ephedra Herb (EH, Ephedrae Herba in Latin) and Cinnamon Bark (CB, Cinnamomi Cortex in Latin) specifically interacted with the central conserved domain (CCD) of G protein, and found that this interaction blocked viral attachment to the cellular receptor CX3CR1 (Fujikane et al., 2022). EH and CB are contained in maoto (ma-huang-tang in Chinese), a traditional herbal medicine that has been used from ancient times in the Far East and has been shown to have clinical efficacy for influenza and influenza-like illness (Nabeshima et al., 2010; Nabeshima et al., 2012; Masui et al., 2017; Fujikane et al., 2022). This *in vitro* anti-viral effect of maoto was also confirmed for RSV infection in a murine model (Nishi et al., 2021).

Antitussive, analgesic, and antipyretic effects of EH have been reported (Tang et al., 2023; Zheng et al., 2023). We have reported that EH extract (EHE) suppressed cancer cell motility and growth through inhibiting c-Met activity (Hyuga et al., 2011; Hyuga et al., 2013; Hyuga et al., 2020; Mori et al., 2023). Unfortunately, adverse reactions such as palpitation, hypertension, insomnia, dysuria, and perspiration can be caused by ephedrine alkaloids through the sympathetic nervous system (Tang et al., 2023; Zheng et al., 2023). To avoid harmful effects, ephedrine alkaloids-free EH extract (EFE) was developed by extracting ephedrine after heating it in water and subjecting it to ion-exchange column chromatography, after which ephedrine alkaloids were below the detection limit (Oshima et al., 2016). Murine and human experiments treated with EFE showed no adverse side effects (Odaguchi et al., 2018; Takemoto et al., 2018). Further, both EFE and EHE displayed c-Met inhibitory, analgesic, anti-influenza virus, and anti-SARS CoV-2 activities, suggesting that the non-alkaloid fraction of EHE contains highly active components other than ephedrine alkaloids (Hyuga et al., 2016; Nakamori et al., 2019; Uema et al., 2023). The active components of EFE were reported to be high-molecular mass condensed tannins (Yoshimura et al., 2020; Uema et al., 2023).

We report here a significant reduction in the viral load of RSV-infected A549 cells treated with EFE, Ephedra Herb extracts from which ephedrine alkaloids are removed. We found that EFE inhibited RSV infection by two pathways: one blocks attachment to the cellular receptor, and the other inhibits replication of RSV in cytoplasm. In the future, EFE, which can be orally administrated, may become an effective and safe therapeutic agent for the treatment of RSV infection.

## Materials and methods

### Cell and virus

A549 cells derived from human lung cancer were purchased from ATCC (Manassas, Virginia, US) and cultured in D-MEM (Fujifilm-Wako, Tokyo, Japan) supplemented with 10% FBS and penicillin/streptomycin at 37°C with 5% CO2. RSV-A2 and -B were also obtained from ATCC. Viruses were grown in the treated A549 cells, and virus-containing culture supernatants were stored at −80°C until use. Virus titers was determined by the plaque-forming unit (PFU) assay as previously described (Fujikane et al., 2022).

### Preparation of anti-viral reagents

EFE was prepared by Kitasato University as previously described (Hyuga et al., 2016). Briefly, EH (200 g) was added to water (2,000 mL), extracted at 95°C for 1 h, filtered, and then the residue was washed with water. The extract was centrifuged, and then the supernatant was concentrated under reduced pressure to obtain the EH extract and passed through the SK-1B ion exchange resin, which was treated with 1 M HCl and water prior to use, and then the resin was washed with water. The unadsorbed fraction was adjusted to pH 5 using 5% NaHCO3aq., and then the solution was evaporated under reduced pressure to obtain EFE (11.8 g). The residual ratios of Ephedrine alkaloids in EFE were less than 1%.

Before experimental use, EFE powder was dissolved and incubated in warm PBS for 1 h. Supernatant was collected after sedimentation at 3,000 ×g, then filtered through a 0.45 μm membrane filter. The dissolved EFE was stored at −80°C until use.

### Cytotoxicity and cell viability assay (CC_50_)

Prepared A549 cells (2.5 × 10^3^) were plated in 96-well plates and treated with EFE (0.5–500 μg/mL) in 10% FBS-containing D-MEM. At 72 h post-treatment, the cell viability was measured with MTS assay, following the manufacturer’s instructions (Promega, Madison, Wisconsin, United States). The value of viability was defined as follows, the metabolic activity of the negative-control wells (with no drug) was set at 100%, and the percentage of reduction was calculated for each EFE concentration. The log versus response logistic nonlinear regression equation in GraphPad Prism10 software (GraphPad Software, San Diego, California, United States) was used to calculate the 50% effective concentration (CC_50_).

### Real-time PCR analysis (IC_50_)

Prepared A549 cells (1 × 10^5^) were plated in 24-well plates and infected with RSV (MOI = 1) mixed with EFE (0–25 μg/mL) at 37°C for 60 min. The virus solution was removed, the cells were washed with PBS, and then incubated at 37°C in the medium for 6 h. The cells were washed with PBS, and total RNA from RSV-infected A549 cells was extracted using ISOGEN II (Nippon Gene, Tokyo, Japan). cDNA was synthesized using a PrimeScript RT reagent kit with gDNA eraser (Toyobo, Osaka, Japan) according to the manufacturer’s instructions. Quantitative PCR was done using the CFX Connect system (Bio-Rad, Hercules, California) as previously described (Fujikane et al., 2022). Briefly, cDNA was amplified using SYBR (Toyobo, Osaka, Japan) with primers for human GAPDH (GCA​CCG​TCA​AGG​CTG​AGA​AC and ATG​GTG​GTG​AAG​ACG​CCA​GT), RSV-A (N region) (CAT​CCA​GCA​AAT​ACA​CCA​TCC​A and TTC​TGC​ACA​TCA​TAA​TTA​GGA​GTA​TCA​A), and RSV-B (P region) (ACG​CTA​CAA​GGG​CCT​CAT​AC and TGC​AAT​GCC​AAA​GTG​CAC​AA), in 40 cycles at 95°C for 15 s and 60°C for 1 min, with analysis by CFX3.1 software (Bio-Rad). The inhibition of viral activity was determined as follows: the metabolic activity of the negative control wells (with no drug) was set at 100%, and the percentage of reduction was calculated for each EFE concentration. The 50% effective concentrations (IC_50_) were determined using the log versus response logistic nonlinear regression equation in GraphPad Prism10 software (GraphPad Software).

### Antibodies

Anti-RSV A2 G protein (ab94966) was purchased from Abcam (Cambridge, United Kingdom). Anti-β-actin (A5316) was obtained from Sigma-Aldrich (St. Louis, MO, United States).

### Western blotting analysis

Cells were harvested in a lysis buffer (100 mM Tris-HCl (pH 6.8), 2% SDS, 20% glycerol, 2% β-mercaptoethanol, and 0.4 mg/mL bromophenolblue), and boiled at 100°C for 20 min. Soluble proteins (25 µg) were separated by SDS-PAGE and transferred to a PVDF membrane (Bio-Rad) by Trans-blot turbo (Bio-Rad). The membrane was immersed with the Can Get Signal PVDF blocking Reagent (Toyobo) for 1 h, then incubated overnight with monoclonal antibodies against RSV-G protein (1:1000, 94966; Abcam, Cambridge, United Kingdom) and β-actin (1:5000, A5316; Sigma-Aldrich, St. Louis, MO, United States). After washing with PBS containing 0.1% tween 20, the membrane was blotted with horseradish peroxidase-conjugated secondary antibody (1:5000, NA931V; cytiva, United States) followed by visualization with a chemiluminescence agent, ECL Prime Western Blotting Detection Reagent (GE Healthcare, Chicago, IL, United States) by LAS-3000 (GE Healthcare).

### Surface plasmon resonance (SPR)

Recombinant C-terminal 6 x His-tagged G-protein was prepared as described previously (Fujikane et al., 2022). CCD peptide was purchased from Cosmo Bio (Tokyo, Japan). The ligands diluted in PBS were immobilized on Sensor Chip NTA (Cytiva, Tokyo, Japan). After washing with PBS, EFE was injected at a flow rate of 30 μL/min for association. A sensergram was obtained using the Biocore J system (Cytiva) and analyzed using Biaevaluation software (Cytiva). All experiments were performed at 25°C.

### Immunofluorescence analysis

RSV-infected A549 cells treated with EFE or left untreated were cultivated for 15 min at room temperature on multichamber dishes fixed with 4% paraformaldehyde diluted in PBS. The cells were treated with 5% BSA in PBS containing 0.1% Tween 20 for blocking and then were incubated at 4°C with anti-G protein antibody (1:500, 94966; Abcam) overnight. After washing with PBS-T, the cells were incubated with Alexa488 conjugated anti-mouse goat antibody (1:1000, A11029; ThermoFisher, Waltham, MA, United States). The dish was mounted with Prolong Diamond antifade mountant with DAPI (ThermoFisher), and the cells were visualized with LSM-710 confocal laser scanning microscopy (Zeiss, Oberkochen, Germany).

### Statistics

Results are shown as the mean value ± standard deviation (error bars) from at least three independent experiments. The statistically significant differences of three or more groups were determined by ANOVA with Dunnett’s *post-hoc* test and those of two group by paired/unpaired Student’s t-test or by Mann-Whitney U test, if the parameters were not distributed normally. IC50 and CC50 were determined using logistic regression testing. By convention, ****p-values  <  0.001, ***p-values  <  0.001, **p-values  <  0.01, and *p-values  <  0.05 versus control. Data were analyzed with GraphPad Prism software (GraphPad Software).

## Results

### An anti-RSV effect of EFE on cultured cells at the binding phase

**FIGURE 1:**
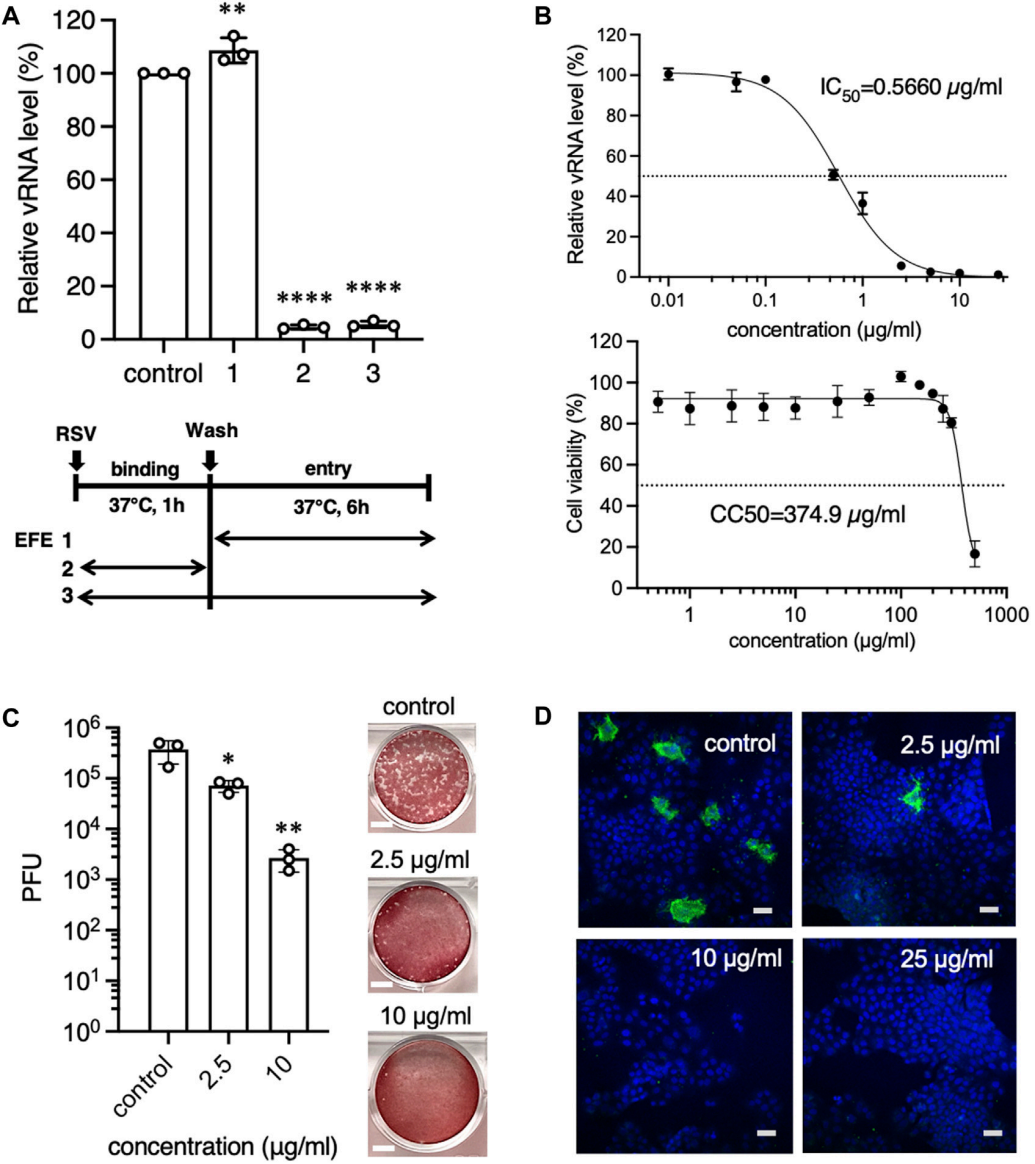
Anti-RSV effects of EFE in cultured cells in the early phase of the virus life cycle. **(A)** A549 cells were inoculated with RSV-A at a moi of 1 for 1 h, followed by washing, and cultured for 6 h. Cells were treated with EFE (2.5 μL/mL) at the entry/replication phase (Shi et al., 2017), binding phase (Young and Smitherman, 2021), or both (Griffin et al., 2020). Viral RNA (vRNA) in the cells was quantified by RT-PCR. **(B)** A549 cells were inoculated with RSV and simultaneously treated with EFE at various concentrations. 6 h later vRNA was quantified and IC50 was calculated (upper panel), then the cytotoxicity of EFE (CC50) was measured (lower panel). **(C)** A549 cells were inoculated with RSV-A at a moi of 1, simultaneously treated with EFE (2.5 and 10 μg/mL) for 1 h and cultured for 24 h. Culture supernatant was examined for viral titers by plaque-forming unit (PFU) assay. Photos in the right panel show the plaques. Scale bars indicate 5 mm. **(D)** Intracellular RSV protein was visualized by immunofluorescence confocal microscopy. Cells were inoculated with RSV, simultaneously treated with various concentrations of EFE, and cultured for 24 h. Green foci indicate RSV G protein. Nuclei were stained blue with DAPI. The numbers in the upper right corner represent drug concentrations. Scale bars indicate 20 µm. **(A–C)** show individual values and mean ± error bars SD (*n* = 3). *p < 0.05, **p < 0.01, and ****p < 0.0001 versus control.

In our previous study, we found that maoto components, extracts of EH and CB, specifically interacted with viral G protein to block viral attachment to the cellular receptor, resulting the reduction of the viral load. As with EHE, we first assessed if EFE would have the same anti-viral effects on cultured A549 cells infected with RSV (subtype A2) for 6 h by real-time (RT) PCR. We treated cells with 2.5 μg/mL EFE simultaneously with RSV inoculation at the binding phase or the subsequent 6-h period after washing out residual virions at the entry/replication phase (Figure 1A). We found that the viral RNA level was significantly decreased when EFE was present at the binding phase, whereas it was not when EFE was present at the entry/replication phase, showing that EFE had an anti-viral effect on cultured cells in the early phase of the virus life cycle. This result is the same as that of a previous study of maoto (Fujikane et al., 2022), suggesting that components other than the ephedrine alkaloids in EHE have the capacity to block infectivity with RSV. Next, we assessed the anti-viral activity and cytotoxicity of EFE against A549 cells. The IC50 and CC50 of EFE were 0.566 μg/mL and 374.9 μg/mL, respectively, and the selection index calculated from them was high at 662 (Figure 1B). The IC50 and CC50 of EFE were about same as those of EHE (Fujikane et al., 2022). We also found that EFE had an anti-viral effect on RSV subtype B, although the IC50 against RSV subtype B was slightly high at 1.125 μg/mL (Supplementary Figure S1).

We confirmed the anti-viral activity of EFE by a plaque-forming unit (PFU) assay that determined if the infective progeny viruses were present in the supernatant 24 h post infection (Figure 1C). The viral titer of the supernatant from infected cells treated with EFE at the binding phase remarkably decreased in a dose-dependent manner. We next visualized replicated viral components in host cells by immunofluorescence confocal microscopy (Figure 1D). Cells with translated viral G proteins (green fluorescence) were observed in the cytoplasm 24 h post-RSV infection, however, the presence of EFE at the binding phase was related to decreased RSV-positive cells 24 h post-RSV infection, in a dose-dependent manner.

### Direct inhibitory effect of EFE on RSV

**FIGURE 2:**
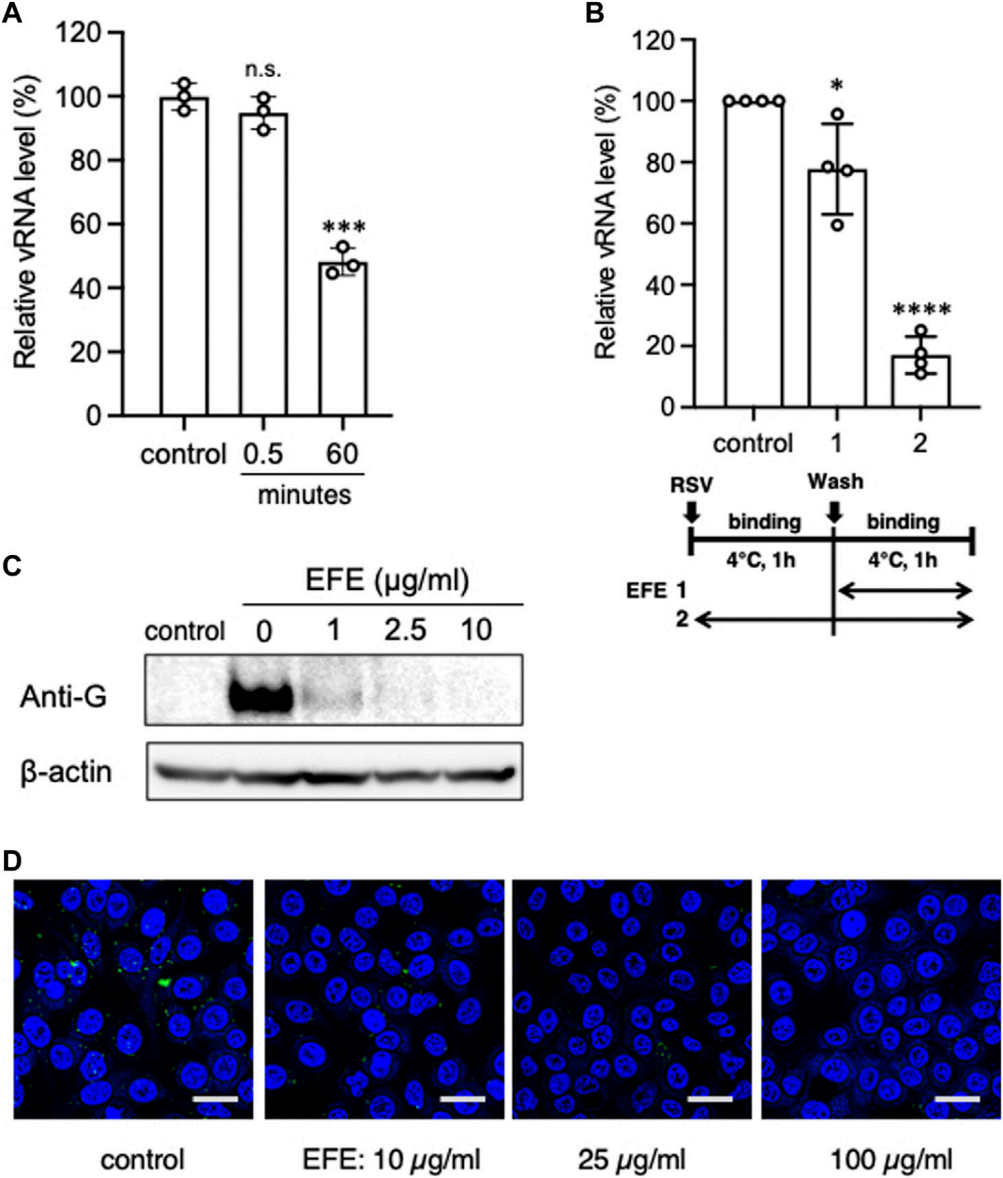
Direct inhibitory effect of EFE on RSV. **(A)** A high concentration of RSV suspension was incubated with EFE (10 μg/mL) for 0.5 or 60 min, then A549 cells were inoculated with the treated RSV (moi:1) and cultured for 6 h (EFE concentration < 0.5 μg/mL), followed by quantification of vRNA. **(B)** Cell surface vRNA on A549 cells was quantified after treatment with 2.5 μL/mL EFE at the binding phase (Shi et al., 2017) or entry/replication phase (Young and Smitherman, 2021) on ice. **(C)** Cell surface viral protein (G protein) was measured by western blotting after treatment at various concentrations (0, 1, 2.5, and 10 μg/mL) of EFE on ice. **(D)** Cell surface viral G protein was visualized by immunofluorescence confocal microscopy. Green foci indicate RSV G protein. Nuclei were stained blue with DAPI. Scale bars indicate 20 µm. **(A,B)** show individual values and mean ± error bars SD (**(A)** *n* = 3, **(B)** *n* = 4). *p < 0.05, ***p < 0.001, and ****p < 0.0001 versus control. n.s: not significant.

We hypothesized that EFE would interact with viral spike proteins to block attachment to host cells, as did EH extract. To test for potential effects of EFE on virions, a high-concentration of RSV was incubated with EFE at a concentration of 10 μg/mL for 0.5 min or 60 min, A549 cells were infected with the treated virions at a multiplicity of infection (moi) 1, then culturing was done for 6 h (final EFE concentration <0.5 μg/mL) (Figure 2A). A significant decrease in the viral RNA level was observed when virions were exposed to EFE for 60 min, suggesting a direct effect of EFE on viral spike proteins. Virions were bound to cell surface receptors at low temperatures without entering the cytoplasm after the viral inoculation, because cellular metabolism is inactive. We further examined the amount of viral RNA and protein on the cell surface to test whether EFE could inhibit viral attachment to the cell surface at low temperatures. EFE treatment significantly reduced the cell surface viral RNA level when EFE treatment was performed at the binding phase (Figure 2B). RSV G protein on the cell surface was also reduced in a dose-dependent manner (Figure 2C). We confirmed this effect by immunofluorescence confocal microscopy, which showed that EFE reduced cell surface green foci (RSV G protein) dose-dependently (Figure 2D). These results suggest that EFE components interact with RSV spike protein to block attachment to cell surface receptors.

### Interaction of EFE components with RSV G protein

**FIGURE 3:**
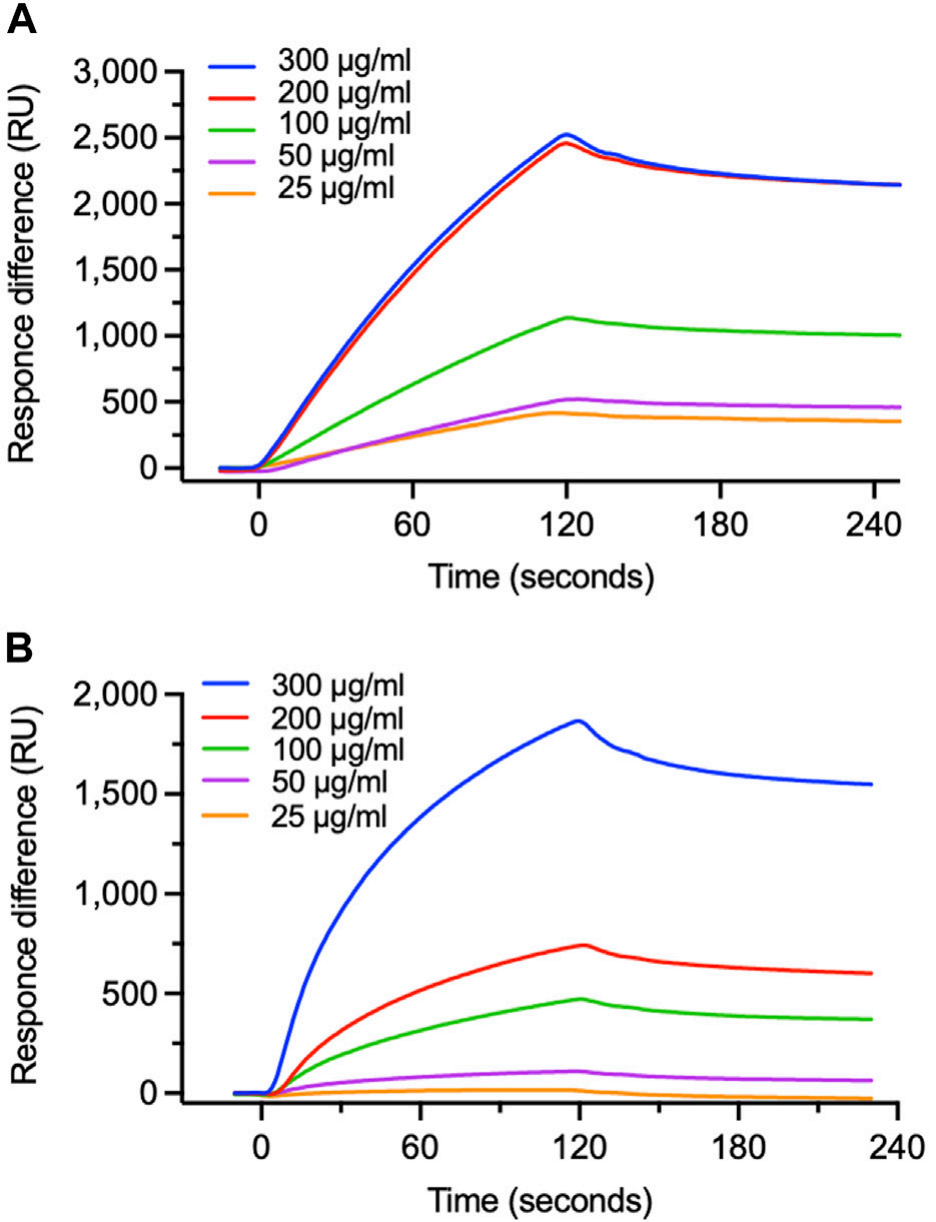
Specific binding of EFE to viral G protein. **(A)** Binding of EFE to RSV G protein immobilized on a sensor chip was measured by surface plasmon resonance (SPR). Five different concentrations (25, 50, 100, 200, and 300 μg/mL) of EFE were injected. **(B)** Binding of EFE to synthesized CCD peptide, the epitope part of G protein, was measured by SPR. Five different concentrations (25, 50, 100, 200, and 300 μg/mL) of EFE were injected.

We focused on the G attachment protein which has CX3C motif on the central conserved domain (CCD) for the epitope to receptor CX3CR1 (Johnson et al., 2015). We then used surface plasmon resonance (SPR) to examine whether EFE would chemically interact with RSV G protein and CCD (Figure 3). Dose-dependent binding was observed when we injected various concentrations of EFE over G protein-immobilized surfaces; moreover, the analyte remained on G protein-immobilized surfaces after washing, suggesting strong interaction between EFE and G protein (Figure 3A). We next tested whether EFE would directly interact with synthesized CCD. SPR analysis showed the dose-dependent binding of EFE with CCD (Figure 3B). These results suggest that EFE interacts with CCD on the RSV G protein to block the attachment to the cellular receptor CX3CR1.

### Another anti-RSV effect of EFE on cultured cells at the entry/replication phase

**FIGURE 4:**
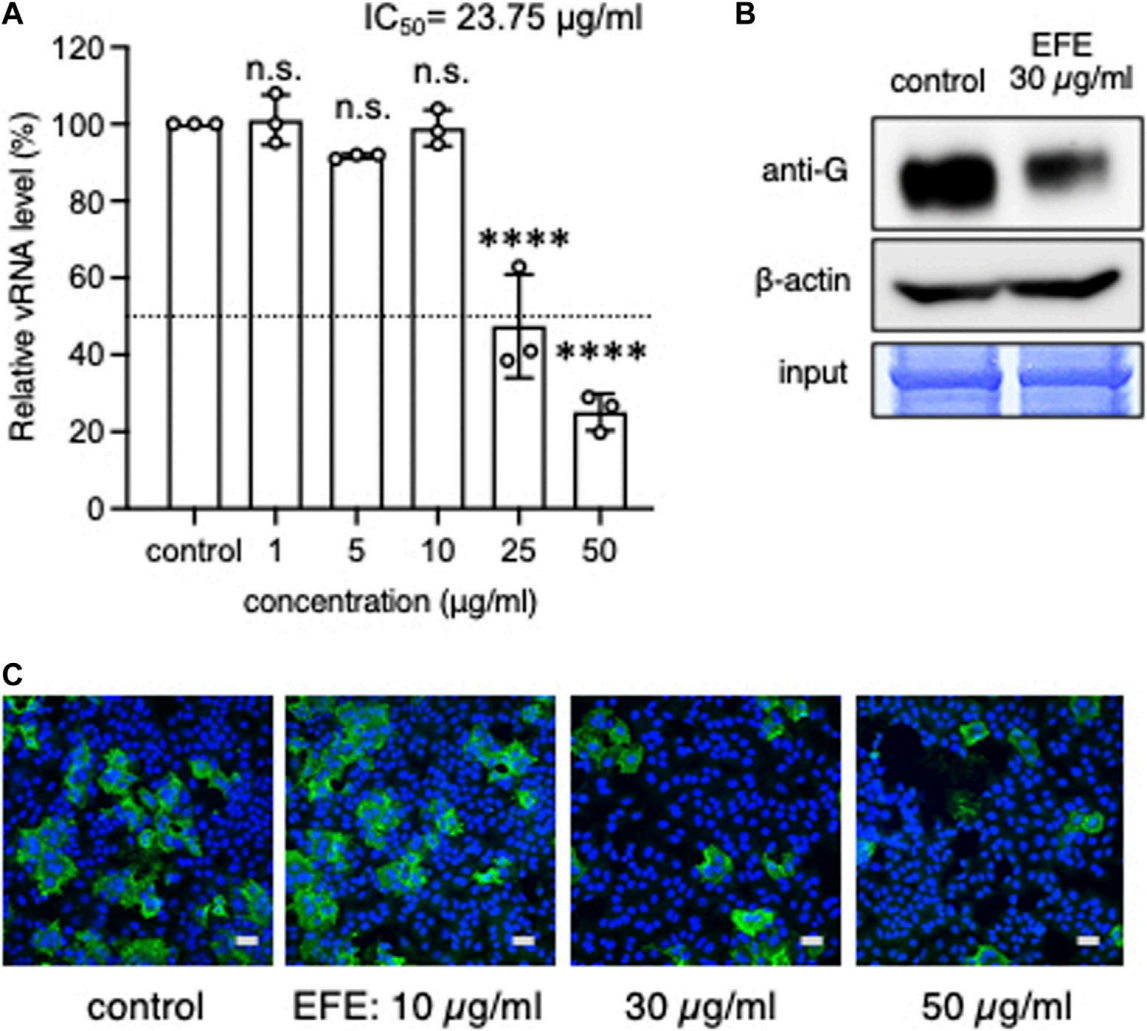
Anti-RSV effects by higher concentration of EFE at the entry/replication phase. **(A)** A549 cells were inoculated with RSV at a moi of 1 for 1 h, the extracellular virions were washed out, and then cultured with various concentrations (0, 1, 5, 25, and 50 μg/mL) of EFE at the entry/replication phase for 6 h. Viral RNA in cultured cells was quantified by RT-PCR. The results show individual values and mean ± error bars SD (*n* = 3). ****p < 0.0001 versus control. n.s: not significant. **(B)** Inoculation of RSV to A549 cells for 1 h was followed by subsequent administration of EFE (30 μg/mL), then cultured for 24 h. Western blotting analysis was performed for translated G protein. **(C)** Inoculation of RSV (moi:1) to A549 cells for 1 h was followed by subsequent administration of 30 μg/mL EFE, then cultured for 24 h. Intracellular viral G protein was visualized by immunofluorescence confocal microscopy. Green foci indicate RSV G protein. Nuclei were stained blue with DAPI. Scale bars indicate 20 µm.

The life cycle of RSV is 6–8 h from attachment to budding. EFE (2.5 μL/mL) did not block the replication of RSV at the entry/replication phase of the culture (Figure 1A). We next sought to determine if a higher concentration of EFE would inhibit the replication of RSV at the entry/replication phase. We added various concentrations of EFE to the cell culture, resulting in 25 μg/mL or more concentrations of EFE inhibited viral replication by RT-PCR. There was no cytopathic effect at this EFE concentration (Figure 4A). Western blotting analysis also showed a decrease in the RSV G protein level when cells were treated with 30 μg/mL EFE 24 h post infection (Figure 4B). Visualization of the effects of EFE at the entry/replication phase was done using immunofluorescence confocal microscopy (Figure 4C). A reduction of the number of virus-positive cells was observed when treated with 30 μg/mL or more EFE.

**FIGURE 5:**
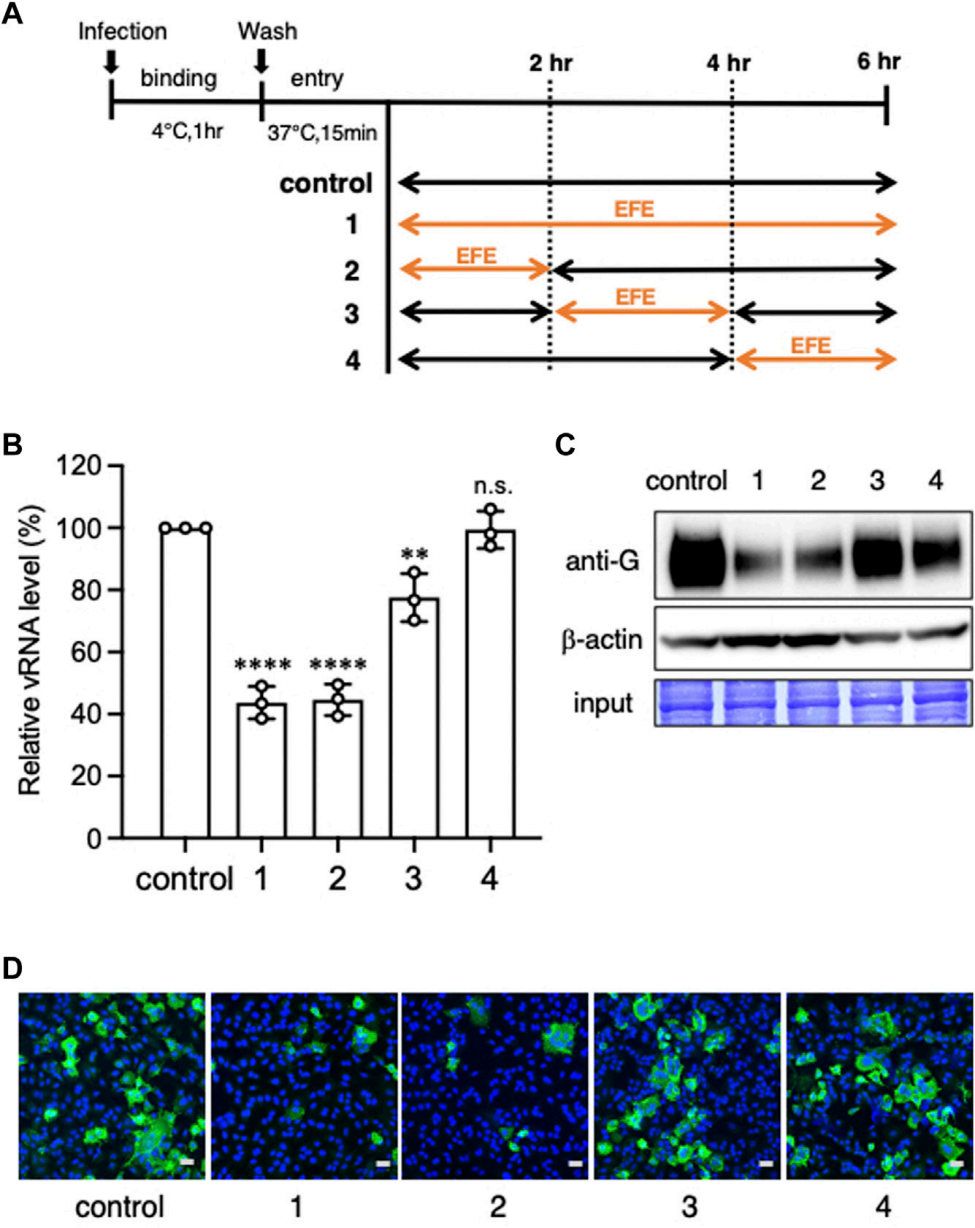
Elucidation of the timing at which EFE showed its anti-viral effect. **(A)** Schema of the culture schedules in Experiments **(B–D)**. A549 cells were inoculated with RSV (moi:1) to bind cell surface at 4°C for 1 h (binding phase), followed by incubation at 37°C for 20 min for the entry of virions, then cultured for 6 h at 37°C. Exposure of EFE (30 μg/mL) to cells was divided into the following phases, 0–6 (Shi et al., 2017), 0–2 (Young and Smitherman, 2021), 2–4 (Griffin et al., 2020), and 4–6 h (Tang et al., 2019). **(B)** Viral RNA in the cells was quantified by RT-PCR. The culture schedule is illustrated in the lower panel. The results show individual values and mean ± error bars SD (*n* = 3). **p < 0.01, and ****p < 0.0001 versus control. n.s: not significant. **(C)** Intracellular viral G protein was measured by western blotting. **(D)** Intracellular viral G protein was visualized by immunofluorescence confocal microscopy. Green foci indicate RSV G protein. Nuclei were stained blue with DAPI. Scale bars indicate 20 µm.

To clarify more precisely when EFE acts to inhibit viral replication at the entry/replication phase, we divided it into four phases, 0–2, 0–6, 2-4, and 4–6 h (Figure 5A). We found that the inhibition of viral replication by 30 μg/mL EFE was at the earliest phase (0–2 h) at both the viral RNA (Figure 5B) and protein levels (Figure 5C). This was confirmed by immunofluorescence confocal microscopy (Figure 5D), which showed a reduction of intracellular G protein at the earliest phase (0–2 h). These results indicate that a higher concentration of EFE (30 μg/mL or more) can provide an anti-viral effect even after virus entry into host cells, especially in the early phase (0–2 h) of the virus life cycle.

## Discussion

We report here that EFE, which includes Ephedra Herb extract from which Ephedrine alkaloids have been removed, had a remarkable anti-viral effect in *in vitro* experimental infection with RSV. We recently reported that maoto, a traditional Japanese herbal medicine, inhibited RSV infectivity in both *in vitro* and *in vivo* experiments (Fujikane et al., 2022). EH extract is one of the crude drugs contained in maoto, and the adverse effects of ephedrine alkaloids, major compounds in maoto, are of concern in its clinical use, especially for patients with cardiovascular diseases and for frail, older patients. EFE was found to be effective, as are maoto and EHE, against RSV infection, indicating that compounds without ephedrine alkaloids are responsible for the anti-RSV effect.

There are at least two possible mechanisms for the anti-RSV effect of EFE. First, EFE can block the attachment of RSV to the cellular receptor, resulting the inhibition of RSV entering the cytoplasm. The CCD of the RSV G spike protein has recently been targeted by therapeutic and prophylactic monoclonal antibodies (Tripp et al., 2018; Anderson et al., 2021). We have confirmed in the present study that EFE can interact with G spike protein and CCD, a main site of viral attachment. Our previous report suggests that the CX3C motif in CCD, the epitope for the receptor CX3CR1, is the main target for EH. Although we did not examine binding to the CX3C motif in the present study, a similar binding mechanism is possible for EFE. Second, EFE was most effective within 2 h after viral inoculation (Figure 5), which suggests that it inhibits the RNA replication of RSV in the early phase of the virus life cycle, even though the viruses may pass through the first barrier of the cell membrane. RSV has a negative-sense, single-stranded genome made of RNA from which a positive-sense anti-genome is synthesized in the cytoplasm by RNA-dependent RNA polymerase (RdRp) encoded in viral RNA just after cell entry (Te Velthuis and Fodor, 2016). RdRp then synthesizes a copy of the genome using the antigenome as a template. We hypothesize that EFE interacts with RdRp to inhibit the synthetization of the genome and/or anti-genome. We are currently doing a study to prove this hypothesis. These above possible mechanisms of EFE are thought to act together to eliminate RSV at different sites during the virus life cycle. There may be other mechanisms. Ephedrannin B showed anti-inflammatory and anti-viral activity in cultured cells infected with RSV in tests that regulated the MAPK/NF-κB signaling pathway (Hou et al., 2020).

Maoto is a traditional Japanese herbal medicine, ‘Kampo,’ that has long been used in the Far East. EH is contained in maoto and many other Kampo formulae and is known to be effective in ameliorating cough, fever, asthma, and pain. As previously reported, EFE exerts analgesic, anti-influenza, and anti-cancer effects in the same manner as EHE (Hyuga et al., 2016; Nakamori et al., 2019; Uema et al., 2023). The main active compound was found to be high-molecular mass condensed tannin from the H_2_O fraction of EFE, with a weight-average molecular weight >45,000 (Yoshimura et al., 2020). For the anti-influenza effect, we and Mantani et al. found that EH extract can block viral fusion through the inhibition of endosomal acidification (Mantani et al., 1999; Masui et al., 2017) that is carried out by vacuolar-type ATPase. EH extract may inhibit the function of vacuolar-type ATPase, which blocks the intake of hydrogen ions into endosomes. Because RSV does not utilize endosomes during infection, but rather fuses directly with the cell membrane before moving into the cytoplasm, the inhibitory mechanism through endosome acidification would be less effective for RSV infection. However, it is possible that the high-molecular mass condensed tannin in EFE interacts with RSV G protein and/or RdRp to inhibit infection and the propagation of RSV. There are likely several additional candidates besides ephedrannin B and high-molecular mass condensed tannin. However, identification of these compounds and exploring their biological functionalities pose formidable challenges and will be a future research endeavor. It is interesting that the crude ingredients in Kampo medicines contain thousands of compounds, some of which may work in concert to inhibit viral infection, such as by RSV and influenza (Nishi et al., 2017). EFE is well tolerated because the toxic effect of Ephedrine alkaloids is eliminated. Once the anti-viral mechanism is elucidated, EFE will become clinically applicable as an effective medicine for oral use against multiple viral diseases.

## Conclusion

EFE, an Ephedra Herb extract from which Ephedrine alkaloids have been excluded, is a crude drug with pharmacologically active effects: analgesic, anti-influenza, and anti-cancer: as does EH extract. We show here that EFE had a remarkable anti-RSV effect on *in vitro* experimental infection. Multiple mechanisms to avoid the infection process were considered, such as blocking virion-cell attachment and viral RNA replication. EFE may be attractive as an oral anti-RSV medicine, especially for children suffering from RSV infection, after undergoing further clinical trials to clarify its anti-viral effects.

## Data Availability

The raw data supporting the conclusions of this article will be made available by the authors, without undue reservation.

## Supplementary material

SUPPLEMENTARY FIGURE 1 Anti-viral effects of EFE on RSV-B. A549 cells were inoculated with RSV-B and simultaneously treated with EFE at various concentrations. Six hours later, intracellular vRNA was quantified and IC50 was calculated supplementary-material.
