# Supplementary figures and images for: Antiviral effect of alkaloids-free Ephedra Herb extract on respiratory syncytial virus infection
Source: Front Pharmacol. 2024 Jul 5;15:1410470. doi: 10.3389/fphar.2024.1410470 (PMC11257991; doi:10.3389/fphar.2024.1410470)

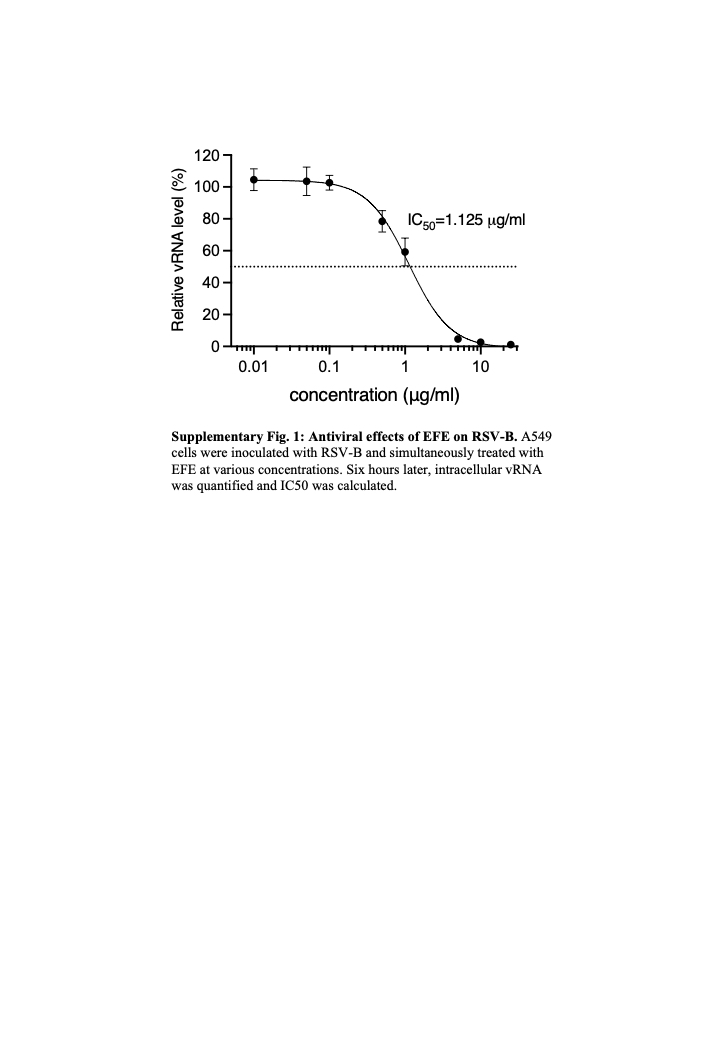

Supplement: Supplementary file 1 [file Image1.jpg]
